# Association between Nrf2 and CDKN2A expression in patients with end-stage renal disease: a pilot study

**DOI:** 10.18632/aging.103685

**Published:** 2020-07-13

**Authors:** Keiichi Sumida, Zhongji Han, Ankur A. Dashputre, Praveen K. Potukuchi, Csaba P. Kovesdy

**Affiliations:** 1 Division of Nephrology, Department of Medicine, University of Tennessee Health Science Center, Memphis, TN 38163, USA; 2 Institute for Health Outcomes and Policy, College of Graduate Health Sciences, University of Tennessee Health Science Center, Memphis, TN 38163, USA; 3 Nephrology Section, Memphis VA Medical Center, Memphis, TN 38104, USA

**Keywords:** aging, CDKN2A, end-stage renal disease, inflammation, Nrf2

## Abstract

Patients with end-stage renal disease (ESRD) display phenotypic features of premature biological aging, characterized by disproportionately high morbidity and mortality at a younger age. Nuclear factor erythroid 2-related factor 2 (Nrf2) activity, a master regulator of antioxidative responses, declines with age and is implicated in the pathogenesis of age-related disorders; however, little is known about the association between Nrf2 and premature biological aging in ESRD patients. In a cross-sectional pilot cohort of 34 ESRD patients receiving maintenance hemodialysis, we measured the expression of Nrf2 and cyclin-dependent kinase inhibitor 2A (CDKN2A, or p16^INK4a^, a biomarker of biological aging) genes in whole blood and examined the association of Nrf2 with CDKN2A expression, using Spearman’s rank correlation and multivariable linear regression models with adjustment for potential confounders. There was a significant negative correlation between Nrf2 and CDKN2A expression (rho=-0.51, *P*=0.002); while no significant correlation was found between Nrf2 expression and chronological age (rho=-0.02, *P*=0.91). After multivariable adjustment, Nrf2 expression remained significantly and negatively associated with CDKN2A expression (*β* coefficient=-1.51, *P*=0.01), independent of chronological age, gender, race, and diabetes status. These findings suggest a potential contribution of Nrf2 dysfunction to the development of premature biological aging and its related morbidities in ESRD patients.

## INTRODUCTION

End-stage renal disease (ESRD) is a condition characterized by a disproportionately high risk of morbidity and mortality, which is almost exclusively seen at a much younger age [1, 2]. This distinct nature of ESRD closely resembles the phenotypic features of premature biological aging, which, in contrast to chronological aging, is characterized by progressive loss of physical and neurological functions, energy balance, and homeostatic mechanisms, often in combination with various organ disorders such as muscle wasting, osteoporosis, vascular calcification, and cardiovascular disease (CVD) [3, 4]. Increased oxidative stress and persistent systemic inflammation induced by several ESRD-related factors (e.g., uremic toxins, bio-incompatibility of dialysis membranes, intravenous iron treatment, and activation of the renin–angiotensin system) have been implicated in the pathogenesis of premature biological aging and multiple age-related comorbidities commonly observed in this population [3, 5].

The nuclear factor erythroid 2-related factor 2 (Nrf2), a transcription factor regulating the expression of an array of cytoprotective genes, has emerged as a key molecule in the adaptive stress response mechanisms against oxidative stress and inflammation [6–10]. In the nucleus, the Nrf2 interacts with small musculoaponeurotic fibrosarcoma (Maf) and co-activator proteins, and activates transcription of Nrf2 target genes by binding to the antioxidant response elements (ARE) in their promoter regions [11, 12]. This Nrf2 signaling leads to the upregulation of various antioxidant and phase 2 detoxifying enzymes, such as NAD(P)H: quinone-oxidoreductase-1 (NQO1), γ-glutamyl cysteinyl synthetase (γ-GCS), and hemeoxygenase-1 (HO-1), which exert antioxidative and anti-inflammatory effects [13–15]. The dysregulation of Nrf2 activation therefore makes cells more sensitive to a variety of stressors, which in turn promotes several key pathological features associated with aging, such as genomic instability, loss of proteostasis, and mitochondrial dysfunction (a.k.a. the hallmarks of aging) [16]. Growing evidence indicates that Nrf2 dysfunction contributes to the development and progression of age-related pathologies, including neurodegeneration, cardiovascular disorders, and cancer [17–20]. Furthermore, a few recent studies have demonstrated reduced expression of Nrf2 in patients with ESRD, potentially mediated in part by chronic systemic inflammation and uremic toxins [21–23].

At a cellular level, aging is associated with accumulation of senescent cells, characterized by an irreversible cell-cycle arrest and increased expression of cyclin-dependent kinase inhibitor 2A (CDKN2A, a.k.a. p16^INK4a^) [24–26], along with several other biological features such as stem cell exhaustion, systemic klotho deficiency, and telomere attrition [27]. As supported by recent evidence indicating the association between senescent cell accumulation and loss of functional capacity in organs [28, 29], CDKN2A is currently considered a validated biomarker of biological aging superior to telomere length that has long been recognized as a relevant measure of aging [25, 30–32]. Recently, a few studies have revealed the biological link between increased CDKN2A expression and Nrf2 dysfunction in age-related pathologies, such as cerebrovascular disorders [33, 34].

Given the unique phenotypic features of increased oxidative stress and premature biological aging in patients with ESRD, it seems plausible that reduced expression of Nrf2 is mechanistically involved in the development of their accelerated biological aging along with increased CDKN2A expression. However, little is known about the association between Nrf2 and CDKN2A expression in the ESRD population. In this pilot study, we therefore aimed to measure the expression of Nrf2 and CDKN2A genes in whole blood of patients with ESRD that were receiving maintenance hemodialysis (HD) and examine the association between Nrf2 and CDKN2A expression in these patients.

## RESULTS

### Baseline characteristics

Patients’ baseline characteristics in the overall cohort and stratified by median Nrf2 expression level are presented in Table 1. Overall, the mean age was 62.6±9.8 years; 52.9% of patients were male; 70.6% were African American; and 70.6% were diabetic. The mean Nrf2 gene expression levels in patients with lower and higher median Nrf2 expression were 6.2±0.5 and 8.1±0.2 (x10^-2^, relative expression quantity), respectively. Compared to patients with higher Nrf2 expression, those with lower Nrf2 expression had significantly higher levels of hemoglobin (11.1±0.9 vs. 10.6±1.5 g/dL, *P*<0.01) and CDKN2A expression (median relative expression quantity 0.14 vs. 0.09, *P*<0.01). No statistically significant differences were observed in demographics, vascular access type, comorbidities, and inflammatory markers between the two groups (Table 1).

**Table 1:** Baseline patient characteristics overall and stratified by median Nrf2 level.

**Characteristics**	**Total (N = 34)**	**Lower Nrf2 (n =17)**	**Higher Nrf2 (n = 17)**	***P***
Age (years)	62.6 ± 9.8	63.4 ± 9.9	61.9 ± 10.0	0.58
Male sex	18 (52.9)	8 (47.0)	10 (58.8)	0.49
Race				0.72
White	8 (23.5)	5 (29.4)	3 (17.7)	
African American	24 (70.6)	11 (64.7)	13 (76.5)	
Others	2 (5.9)	1 (5.9)	1 (5.9)	
Dialysis vintage (years)	5.3 ± 2.8	5.1 ± 3.3	5.6 ± 2.3	0.43
Vascular access type				0.82
Arteriovenous fistula	24 (70.6)	12 (70.6)	12 (70.6)	
Arteriovenous graft	5 (14.7)	3 (17.6)	2 (11.8)	
Catheter	5 (14.7)	2 (11.8)	3 (17.6)	
Charlson Comorbidity Index	5.9 ± 1.7	5.9 ± 1.8	5.8 ± 1.7	0.78
Diabetes mellitus	24 (70.6)	10 (58.8)	14 (82.4)	0.13
Ischemic heart disease	6 (17.7)	3 (17.7)	3 (17.7)	-
Congestive heart failure	4 (11.8)	2 (11.8)	2 (11.8)	0.29
Cancer	0 (0)	0 (0)	0 (0)	-
Laboratory markers				
Hemoglobin (g/dL)	10.9 ± 1.2	11.1 ± 0.9	10.6 ± 1.5	0.025
Albumin (g/dL)	4.0 ± 0.3	4.0 ± 0.3	4.0 ± 0.3	0.56
LPS (EU/mL)	0.11 [0.07, 0.15]	0.11 [0.08, 0.15]	0.11 [0.05, 0.13]	0.62
CRP (mg/L)	1.8 [0.9, 3.7]	1.3 [0.7, 3.7]	2.1 [1.3, 3.6]	0.20
TNF-α (pg/mL)	9.4 ± 3.0	8.9 ± 2.8	9.8 ± 3.2	0.33
IL-6 (pg/mL)	3.2 [2.2, 7.4]	3.0 [2.0, 8.9]	3.2 [2.3, 7.4]	0.87
MCP-1 (ng/mL)	165.3 ± 53.8	167.2 ± 60.5	163.4 ± 47.9	0.97
CDKN2A expression (x10^-1^, RQ)	1.0 [0.8, 1.4]	1.4 [0.9, 1.8]	0.9 [0.6, 1.1]	<0.01
Nrf2 expression (x10^-2^, RQ)	7.2 ± 1.1	6.2 ± 0.5	8.1 ± 0.2	<0.01

*Note*: Data are presented as number (percentage), mean ± SD, or median [IQI].

Abbreviations: CDKN2A = cyclin dependent kinase inhibitor 2A; CRP = C-reactive protein; IL-6 = interleukin-6; IQI = interquartile interval; LPS = lipopolysaccharide; MCP-1 = monocyte chemoattractant protein-1; Nrf2 = nuclear factor-erythroid 2-related factor 2; RQ = relative quantity; SD = standard deviation; TNF-α = tumor necrosis factor alpha

### Association between Nrf2 and CDKN2A expression

**Figure 1:**
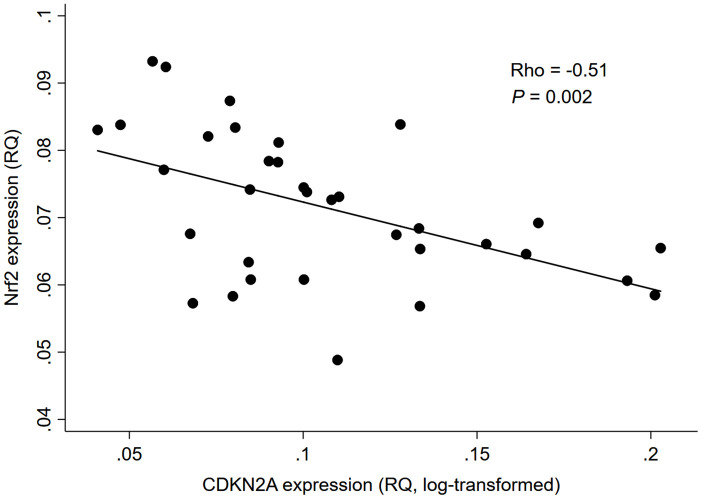
**Correlation between Nrf2 and CDKN2A expression levels in 34 HD patients.** Abbreviation: CDKN2A = cyclin dependent kinase inhibitor 2A, Nrf2 = nuclear factor erythroid 2 related factor 2, RQ = relative quantity.

**Figure 2:**
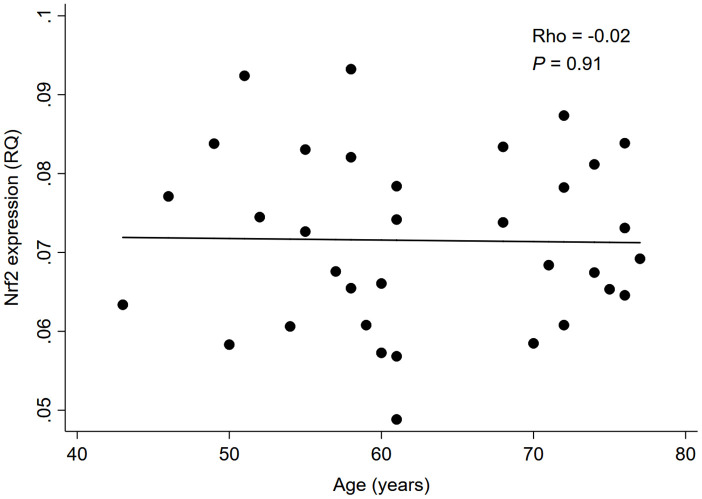
**Correlation between Nrf2 expression level and chronological age in 34 HD patients.** Abbreviation: Nrf2 = nuclear factor erythroid 2 related factor 2, RQ = relative quantity.

As depicted in Figure 1, there was a significant negative correlation between Nrf2 and CDKN2A expression levels in whole blood of 34 HD patients (rho=-0.51, *P*=0.002); while no significant correlation was found between Nrf2 expression level and chronological age (rho=-0.02, *P*=0.91; Figure 2).

Table 2 shows the association between Nrf2 and CDKN2A expression levels using univariable and multivariable linear regression analyses. In the univariable linear regression model, a significant negative association was observed between Nrf2 and CDKN2A expression levels (*β* coefficient=-1.98, *P*=0.002). A statistically significant association with CDKN2A expression was also observed for higher age (*β* coefficient=0.00177, *P*=0.018), male gender (*β* coefficient=0.0300, *P*=0.040 [vs. female]), and African American race (*β* coefficient=-0.0378, *P*=0.031 [vs. white]). After adjustment for variables that were statistically significant in the univariable model (i.e., Nrf2 expression, age, gender, and race), only Nrf2 expression was found to be significantly associated with the CDKN2A expression level (*β* coefficient=-1.84, *P*=0.001, in Model 1). The negative association between Nrf2 and CDKN2A expression remained statistically significant even after additionally accounting for the variable significant at *P*<0.1 in the univariable analysis (i.e., diabetes) (*β* coefficient=-1.51, *P*=0.010, in Model 2; Table 2). Similar results were observed when we replaced diabetes with inflammatory markers (i.e., C-reactive protein [CRP], tumor necrosis factor alpha [TNF-α], interleukin-6 [IL-6], and monocyte chemoattractant protein-1 [MCP-1]) or serum albumin concentration.

**Table 2:** Association between Nrf2 and CDKN2A expression levels in univariable and multivariable models.

**Characteristics**	**Univariable**			**Model 1**			**Model 2**		
	***β***	**Std. Err.**	***P***	***β***	**Std. Err.**	***P***	***β***	**Std. Err.**	***P***
Nrf2 expression (RQ)	-1.98	0.598	0.002	-1.84	0.520	0.001	-1.51	0.547	0.010
Age (years)	0.00177	0.000709	0.018	0.000774	0.000794	0.34	0.00144	0.000878	0.11
Male (vs. female)	0.0300	0.0140	0.040	0.0239	0.0141	0.10	0.0162	0.0145	0.28
Race (vs. white)									
African American	-0.0378	0.0167	0.031	-0.0233	0.0153	0.14	-0.00922	0.0172	0.60
Others	-0.0452	0.0324	0.17	-0.0526	0.0267	0.059	-0.0633	0.0268	0.026
Dialysis vintage (year)	-0.00267	0.00264	0.32						
Vascular access (vs. AVF)									
AVG	0.0359	0.0209	0.87						
Catheter	-0.0328	0.0209	0.13						
Charlson Comorbidity Index	0.0288	0.00442	0.52						
Diabetes	-0.0311	0.0155	0.053				-0.0268	0.0167	0.12
Ischemic heart disease	0.0205	0.0193	0.295						
Laboratory markers									
Hemoglobin (g/dL)	0.00126	0.00632	0.84						
Albumin (g/dL)	-0.0277	0.0241	0.26						
LPS (EU/mL)*	0.0438	0.108	0.69						
CRP (mg/L)*	-0.0174	0.0121	0.16						
TNF-α (pg/mL)	-0.00334	0.00249	0.19						
IL-6 (pg/mL)*	-0.00163	0.0102	0.87						
MCP-1 (ng/mL)	0.000229	0.000135	0.10						

*Values were log-transformed

*Note*: Regression coefficients for each covariate (for a one unit increase in continuous variables) are presented for every one unit increase in log-transformed CDKN2A expression levels.

Model 1 is adjusted for only variables that were statistically significant in univariate analysis (i.e., Nrf2, age, gender, and race); and model 2 is adjusted for the variables in model 1 plus diabetes (i.e., the variable significant at *P* <0.1 in univariate analysis).

Abbreviations: AVF = arteriovenous fistula; AVG = arteriovenous graft; CDKN2A = cyclin dependent kinase inhibitor 2A; CRP = C-reactive protein; IL-6 = interleukin-6; LPS = lipopolysaccharide; MCP-1 = monocyte chemoattractant protein-1; Nrf2 = nuclear factor-erythroid 2-related factor 2; RQ = relative quantity; SD = standard deviation; Std. Err. = standard error; TNF-α = tumor necrosis factor alpha

## DISCUSSION

In this cross-sectional pilot study of 34 ESRD patients receiving maintenance HD, we found that whole blood Nrf2 expression was negatively correlated with CDKN2A expression, but not with chronological age. We also demonstrated that the negative association between Nrf2 and CDKN2A expression was independent of chronological age, gender, race, diabetes status, and inflammatory markers.

With a growing recognition of increased oxidative stress and inflammation as key determinants of aging (a.k.a. “inflammaging”) [35, 36], Nrf2 has recently received special attention to its pivotal role in the development of a wide range of aging phenotypes [18]. Emerging evidence has indicated that the transcriptional activity of Nrf2 declines with age in several tissues and organs such as vasculature [37] and brain [38], and currently the induction of cellular senescence, which is an evolutionarily conserved cellular stress response mechanism, is considered a fundamental aging process mediated by Nrf2 dysfunction [39]. In an animal study investigating age-related changes in the expression of senescence markers, CDKN2A expression was found to be upregulated in cerebral arteries of aged (vs. young) mice, which was further exacerbated by genetic depletion of Nrf2, along with an increase in gene expression of several pro-inflammatory senescence-associated secretory phenotype (SASP) factors (e.g., IL-1β and TNF-α) [33]. A recent experimental study using human epidermal keratinocytes also demonstrated that genetic silencing of Nrf2 enhanced the accumulation of CDKN2A protein in keratinocytes, suggesting the mechanistic involvement of Nrf2 dysregulation in the process of cellular senescence in human epidermis [34].

In line with these previous findings, our results showed for the first time, to the best of our knowledge, that lower Nrf2 expression was associated with higher CDKN2A expression in whole blood of patients with ESRD. Of note, this negative Nrf2-CDKN2A association was independent of chronological age, implying that the age-related morbidities commonly seen in patients with ESRD may not necessarily be a consequence of their chronological aging but rather result from other factors linked to Nrf2 signaling specific to this patient population. In ESRD, over half of the patients are reported to have serologic evidence of an active inflammatory response (a.k.a. “uremic inflammation”) [40, 41], which has also been implicated in the pathogenesis of premature biological aging and age-related comorbidities [3, 25]. In this study, however, there was no significant association between inflammatory markers and CDKN2A, which appear to contradict prior evidence. Although it is possible that the non-significant association with inflammatory markers was merely due to the small sample size of this study, there may be a plausible explanation for this observation. Recently, studies have revealed that Nrf2 lies at the center of a complex regulatory network and is involved in a wide range of cellular processes, including not only redox regulation and pro-inflammatory response, but also DNA repair, mitochondrial function, proteostasis, proliferation, and iron, lipid, carbohydrate, and drug/xenobiotic metabolisms, all of which contribute to cell survival [42]. It is therefore possible that the cellular senescence associated with Nrf2 dysfunction in ESRD is predominantly meditated by the alterations of these latter cellular processes, such as mitochondrial dysfunction and metabolic imbalance, rather than enhanced pro-inflammatory response. Another ESRD-specific factor linked to Nrf2 signaling may be the toxic internal milieu, characterized by the accumulation of a variety of uremic toxins, such as indoxyl sulfate (IS), *p*-cresyl sulfate (*p*-CS), and indole-3-acetic acid (IAA) [43]. A previous experimental study demonstrated that IS, one of the potent uremic toxins, induced endothelial cell senescence by increasing reactive oxygen species production and p53 activity [44]. In another recent study examining the correlation between plasma uremic toxins and Nrf2 expression in peripheral blood mononuclear cells (PBMCs) of HD patients, plasma IS was shown to be negatively correlated with Nrf2 expression in PBMCs [21]. Given these findings and the lack of significant correlation between Nrf2 and chronological age observed in our study, it is possible that Nrf2 dysfunction induced by uremic toxins promotes cellular senescence independently of chronological age, leading to the development of premature biological aging and so-called “age-related” comorbidities in ESRD (as these may not actually be related to chronological age). This may also be supported by a recent finding that high arterial CDKN2A expression was significantly associated with severe arterial vascular calcification, a hallmark of premature aging, in uremic patients undergoing living donor kidney transplantation, independently of their chronological age [45]. The study also demonstrated that the arterial CDKN2A expression increased with the degree of medial calcification and was negatively associated with carboxylated osteocalcin levels, suggesting the pathogenic contribution of impaired vitamin K-mediated carboxylation to premature vascular senescence [45]. Furthermore, increasing evidence indicates that the Nrf2 plays a key role in the process of vascular calcification, partly through its regulatory effect of osteoblastic differentiation [46, 47]. In this context, our findings on the chronological age-independent Nrf2-CDKN2A association in ESRD patients may be of particular value, with several potential clinical and research implications. Although previous studies consistently revealed that the Nrf2 expression declines with age, this may not necessarily apply to the ESRD population. While it is broadly accepted that chronological age is a well-established risk factor for many age-related diseases, biomarkers of biological aging, such as whole blood expression of CDKN2A gene, could also have the potential to serve as novel noninvasive prognostic markers of adverse events associated with age-related diseases (e.g., CVD and mortality) in patients with ESRD. Given the potential contribution of a repressed Nrf2 system to premature biological aging in ESRD, both synthetic (e.g., bardoxolone methyl) [48] and natural nutrigenomic compounds (e.g., sulforaphane) [49] that have been shown to restore Nrf2 expression could be novel therapeutic options against premature morbidity and mortality in patients with ESRD. All of these possibilities may deserve further investigation.

The study results must be interpreted in light of several limitations. Our study sample was small and not representative of patients with ESRD who are heterogeneous with respect to various etiologies and comorbidities. Due to the small sample size, we were unable to fully account for potential confounders. In addition, we cannot eliminate the possibility of unmeasured confounders, such as diet and lifestyle, that might have influenced the association between Nrf2 and CDKN2A expression. Since RNA was prepared from a homogenized whole blood sample, this procedure precludes any knowledge on how the expression varied between different cell types in whole blood, although no data are currently available regarding the expression variation of Nrf2 and CDKN2A genes across different blood cell types of ESRD patients. Furthermore, blood samples in this cohort were collected before the start of dialysis treatment, and thus we could not compare the difference of whole blood Nrf2 expression between pre- and post-dialysis. Lastly, it should also be noted that the gene expression levels of Nrf2 and CDKN2A may vary depending on different tissues or organs. It is therefore important to examine the Nrf2-CDKN2A association in other tissues or organs, such as arteries, muscle, and fat, of patients with ESRD in future studies. Nevertheless, given the non-invasive nature of blood sampling, our study results using whole blood samples would still be of value in terms of potential applications for research and clinical purposes, particularly if our findings are confirmed in other tissue specimens of ESRD patients.

In conclusion, in this cross-sectional pilot study of 34 prevalent HD patients, we found a significant negative association between the whole blood expression of Nrf2 and CDKN2A genes, independent of chronological age. Our findings suggest a potential contribution of Nrf2 dysfunction to the development of premature biological aging and age-related morbidities in patients with ESRD. Further in-depth basic and clinical studies are needed to clarify the underlying pathophysiologic mechanisms for the Nrf2-CDKN2A association and examine if Nrf2-targeted interventions could retard the progression of biological aging and its related complications in ESRD patients.

## MATERIALS AND METHODS

### Study design

This was a prospective study of anonymized samples and statistically de-identified clinical data obtained from a biorepository assembled by DaVita Clinical Research (Minneapolis, MN, USA). Anonymized samples and statistically deidentified data were made available to the researcher for academic research via a grant program called BioReG.

### Study population

The DaVita Clinical Research biorepository comprises blood samples and clinical data from 4,028 individuals with prevalent end-stage renal disease who received hemodialysis at a large dialysis organization (LDO) between May 2011 and October 2013. The biorepository sampling protocol was reviewed and approved by an Institutional Review Board (IRB) (Quorum IRB, Seattle, WA, USA) and patients provided written informed consent prior to the initiation of sample collection. Patients with hemoglobin <8.0 g/dL, who were <18 years of age, who were pregnant, or who had any physical, mental, or medical condition which prohibited the ability to provide informed consent were excluded from participation.

For the present cross-sectional pilot study, we used biospecimens and data at baseline (i.e., first blood sampling date) from 34 HD patients within the repository housed at the University of Tennessee Health Science Center (UTHSC) (UT-DaVita HD cohort; n=978). The study was approved by the IRB of the UTHSC (IRB protocol numbers: 16-04357-XP and 17-05299-XP).

### Biorepository biospecimen and clinical data collection

Under the biorepository study protocol, blood samples were collected from each subject at baseline and, thereafter, every 3 months for up to one year. Pre-dialysis blood samples were collected and processed according to a standardized protocol: specimens were shipped on refrigerated packs on the day of collection to a centralized laboratory, where they were aliquotted and stored at -80°C. Specimens with cause for rejection (e.g., unspun tubes, insufficient volume, or thawed specimens) or that were received >48h from the time of collection were rejected. Anonymized plasma samples were shipped from the centralized laboratory to the researchers on dry ice at -80°C.

Clinical and hemodialysis treatment data for each biorepository subject were collected by the LDO during the course of routine care and were maintained in the LDO electronic health record. Clinical and hemodialysis treatment data were provided to the researchers by DaVita Clinical Research in statistically deidentified form.

### Biomarker measurements

In addition to the variables available from the laboratory measurements obtained during routine care, plasma lipopolysaccharide (LPS) and specific inflammatory markers including CRP, TNF-α, IL-6, and MCP-1 were additionally measured in our pilot study. Plasma LPS levels (EU/mL) were quantified in duplicate using a Pierce LAL Chromogenic Endotoxin Quantitation Kit (ThermoFisher Scientific, Waltham, MA, USA) as per the manufacturer’s protocol. Plasma levels of CRP, TNF-α, IL-6, and MCP-1 were measured and verified using the Magnetic Luminex (Magpix) platform from R&D systems (R&D Systems, Inc., Minneapolis, MN) following the manufacturer’s recommendations [50].

### RNA preparation and gene expression analysis

The PAXgene blood RNA kit (Qiagen, Hilden, Germany) was used to perform total RNA extraction from whole blood samples according to the manufacturer’s recommendations. The RNA samples were reverse transcribed into cDNA using Transcriptor First Strand cDNA synthesis kit (Roche, Basel, Switzerland), and a 500 ng total RNA sample was used for the single strand cDNA synthesis. Gene transcript level was quantified using LightCycler® 480 System (Roche, Basel, Switzerland). TaqMan Gene Expression Assays specific to Nrf2 (Hs00975961_g1, Applied Biosystems, Carlsbad, CA) and CDKN2A (Hs00923894_m1, Applied Biosystems, Carlsbad, CA) were used to determine Nrf2 and CDKN2A expression levels. Relative expression levels of Nrf2 and CDKN2A were normalized against GAPDH (Hs02758991_g1, Applied Biosystems, Carlsbad, CA) [51] and HPRT (Universal Probe Library Human HPRT gene assay, Roche, Basel, Switzerland) [45, 52], respectively. All assays were performed in triplicate, and the comparative threshold cycle (CT) method was used to quantify relative gene expression [53]. To ensure the rigor of our measurements, the intra-assay coefficient of variation for the gene expression levels of Nrf2 and CDKN2A was tested respectively using the TaqMan Gene Expression Assays which demonstrated good reproducibility (3.8% and 5.1% for Nrf2 and CDKN2A, respectively).

### Statistical analysis

Baseline patient characteristics were presented as number (percentages) for categorical variables and mean (standard deviation [SD]) for continuous variables with a normal distribution or median (interquartile interval [IQI]) for those with a skewed distribution. Differences between groups were assessed using the chi-squared test or Wilcoxon rank-sum test, as appropriate. Spearman’s rank correlation (rho) was used to determine the correlations of Nrf2 expression with CDKN2A expression and chronological age. We preformed univariable and multivariable linear regressions to examine the association between Nrf2 and CDKN2A expression levels. Variables with a skewed distribution were treated as log-transformed continuous variables, as appropriate. Given the limited sample size in this pilot study, the following two incremental models were used based on theoretical consideration and data availability: model 1 included variables that were statistically significant in the univariable analysis; and model 2 additionally accounted for the variables that were significant at *P*<0.1 in the univariable analysis. The regression coefficients for each covariate were presented for every one-unit increase in log-transformed CDKN2A expression levels. A threshold of statistical significance was set at the level of *P*<0.05 for all analyses unless otherwise specified. Statistical analyses were conducted in STATA/MP Version 15 (STATA Corporation, College Station, TX).
